# Heart failure drug classes and 30-day unplanned hospital readmission among patients with heart failure in Ethiopia

**DOI:** 10.1186/s40780-023-00320-y

**Published:** 2023-11-27

**Authors:** Birhanu Ayenew, Prem Kumar, Adem Hussein, Yegoraw Gashaw, Mitaw Girma, Abdulmelik Ayalew, Beza Tadesse

**Affiliations:** 1 Department of Adult Health Nursing, College of Health Science, Assosa University, Assosa, Ethiopia; 2 Department of Adult Health Nursing, College of Medicine and Health Science, Wollo University, Dessie, Ethiopia; 3 Department of Pediatric and Child Health Nursing, College of Health Science, Assosa University, Assosa, Ethiopia; 4 Department of Comprehensive Health Nursing, College of Medicine & Health Sciences, Wollo University, Dessie, Ethiopia

**Keywords:** Hospital readmission, Heart failure, Drug therapy, Ethiopia

## Abstract

**Background:**

Drug therapy is a crucial aspect of heart failure management and has been shown to reduce morbidity and mortality in heart failure patients. However, the comparative effects of these drug classes on readmission rates have not been well studied. Therefore, the aim of this study was to examine the association between different classes of heart failure drugs and 30-day readmission rates in patients with heart failure.

**Method:**

A multicenter, hospital-based retrospective cohort design was employed and 572 randomly selected patients with heart failure were included. Data were entered in Epi-data version 4.6 and analyzed with STATA version 17. Kaplan-Meier and log-rank tests were used to estimate and compare survival time. A Cox proportional hazard model was utilized, employing both bi-variable and multi-variable analyses, to examine the effect of predictors on the timing of unplanned hospital readmissions. The strength of the association was assessed using an adjusted hazard ratio (aHR), and statistical significance was declared for p-values < 0.05 and a 95% confidence interval (CI).

**Results:**

In this study, a total of 151 (26.40%) heart failure patients were readmitted within 30 days of discharge. In the multivariate cox proportional hazards analysis being an age (> 65 year) (AHR: 2.34, 95%CI: 1.63, 3.37), rural in residency (AHR: 1.85, 95%CI: 1.07, 3.20), hospital stays > 7 Days (AHR: 3.68, 95%CI: 2.51,5.39), discharge with Diuretics (AHR: 2.37, 95%CI: 1.45, 3.86), and discharge with Beta-Blocker (AHR: 0.48, 95%CI: 0 0.34, 0.69) were identified as independent predictors of unplanned hospital readmission.

**Conclusion:**

Elderly patients, being in rural areas, longer hospital stays, and discharges of patients on diuretics and not on beta-blockers were independent predictors of unplanned hospital readmission. Therefore, working on these factors will help to reduce the hazard of unplanned hospital readmissions, improve patient outcomes, and increase the efficiency of heart failure management.

## Introduction

Heart failure is a global public health issue, affecting millions of people worldwide and resulting in significant morbidity, mortality, and healthcare costs [1]. The prevalence of heart failure is increasing, and it is estimated that by 2030, more than 8 million people in the United States alone will have heart failure [2]. Hospitalizations for heart failure are common and account for a significant portion of healthcare expenditure. However, despite advances in heart failure management, the 30-day readmission rates among heart failure patients remain high, with up to 25% of patients being readmitted within 30 days of discharge [3, 4].

In Africa, heart failure patients lack knowledge about their disease, medications, and side effects. This contributes to poor outcomes, driven by discharge planning issues and increased hospital admissions [5]. Unlike high-income countries, heart failure affects young and middle-aged adults in sub-Saharan Africa, impacting economic growth [6]. Hospital studies indicate that heart failure comprises 9.4–42.5% of medical admissions and 25.6–30.0% of cardiac unit admissions [7].

Reducing readmission rates is a critical goal in heart failure management, as readmissions can result in increased morbidity, mortality, and healthcare costs [8]. Identifying factors that contribute to readmission is crucial in developing strategies to reduce readmission rates. While several patient-related factors, such as comorbidities, and older age have been identified as risk factors for readmission, the effects of heart failure drug classes on readmission rates are not well understood [9, 10].

Drug therapy is a crucial aspect of heart failure management and has been shown to reduce morbidity and mortality in heart failure patients [11]. Several drug classes, including angiotensin-converting enzyme inhibitors (ACEIs), angiotensin II receptor blockers (ARBs), beta-blockers, aldosterone antagonists, and diuretics, are commonly prescribed in heart failure management [12]. However, the comparative effects of these drug classes on readmission rates have not been well studied.

In countries like Ethiopia, emphasis is primarily placed on addressing primary healthcare, with limited focus on quality improvement, despite commitments to Sustainable Development Goal 3.4 [13]. Currently, there is a lack of published research in Ethiopia that comprehensively examines association between heart failure drug classes and 30-day readmission rates in patients with heart failure.

Hence, this study aimed to investigate the association between the use heart failure drug classes and 30-day readmission in patients with heart failure.

## Materials and methods

### Study population

A Multicenter hospital based retrospective follow-up study was conducted in the South Wollo Zone from January 2016 to December 2020, including Boru-Meda General Hospital, Akesta General Hospital, and Mekane-Selam General Hospital. The study included heart failure patients hospitalized between January 2016 and December 2020, who were ≥ 18 years old. However, patients discharged with recorded deaths were excluded to avoid bias.

### Definition

#### Event

The occurrence of readmission within 30 days after hospital discharge from an index admission.

#### Unplanned hospital readmission

Any patient with heart failure who is hospitalized within 30 days of initial discharge, excluding planned readmissions or scheduled follow-up visits, and unavoidable emergencies such as a car accident or combat injury. Unplanned readmissions within this 30-day window are considered relevant events for study analysis.

The diagnosis of heart failure was confirmed through the reference of International Classification of Diseases-11 (ICD-11) codes [14], utilizing information extracted from medical records. For categorizing prescription drugs, we utilized therapeutic classification systems based on drug categories.

#### Follow-up time

From the time of discharge until an event occurred.

#### Censored

Patients who did not readmit within 30 days during the follow-up period.

#### Survival status

The status of heart failure patients at the end of the follow-up period (readmission or censored).

#### Time to readmission

The Time interval from discharge from the hospital till readmission happens.

### Sampling procedure and data collection

The list of patients hospitalized with the index admission of heart failure (HF) was obtained from the Health Management Information System registration. Health Management Information System (HMIS) is an electronic health record system and tool used to record, store, retrieve, and process health and health related data to improve decision-making.

**Fig. 1:**
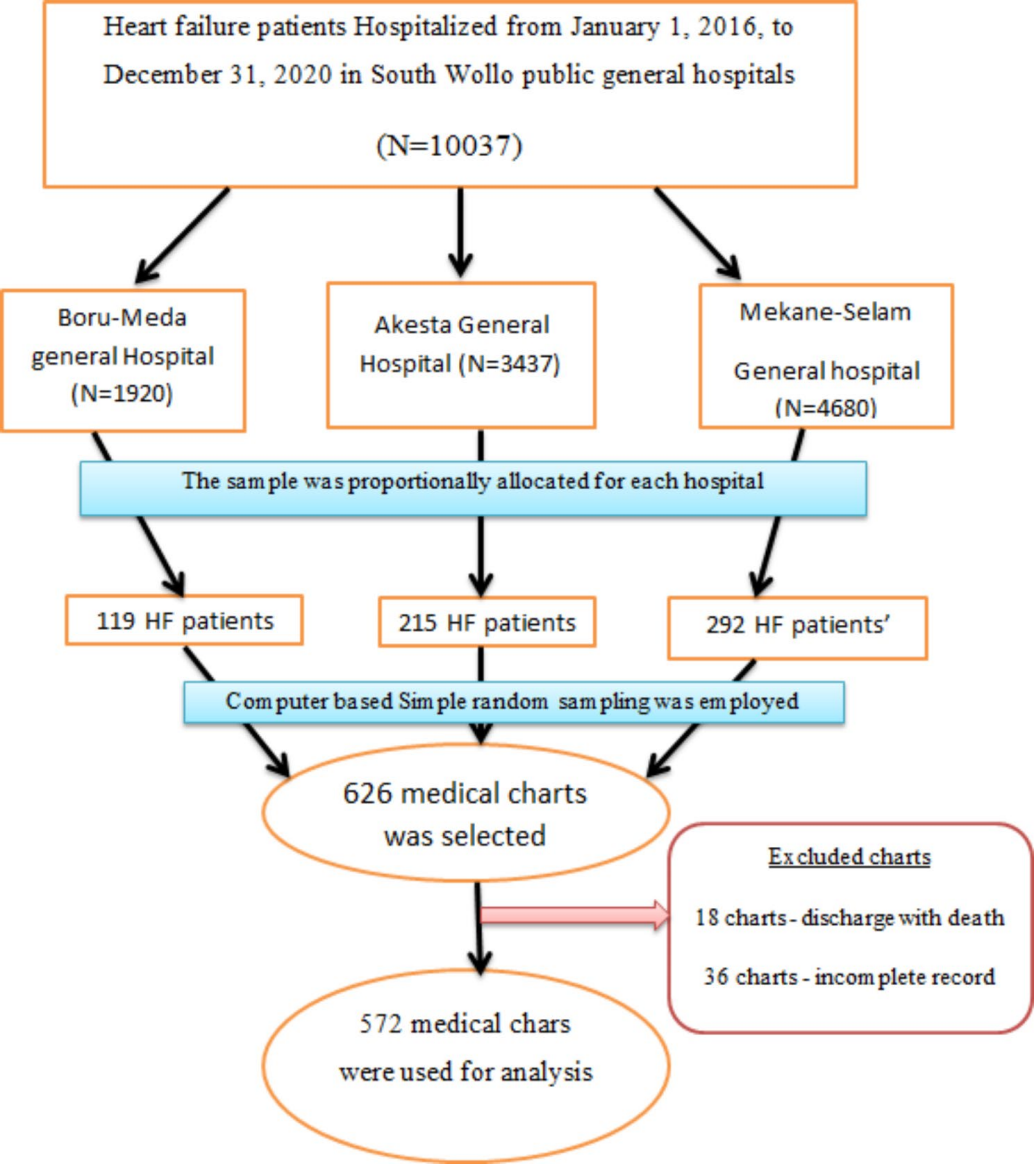
Schematic representation of sample selection among adult heart failure patients at selected South Wollo general hospitals in 2022

During the five-year study period, a total of 10,037 heart failure (HF) patients were enrolled from three different hospitals. To ensure a representative sample, the sample size for the study was distributed proportionally among the three hospitals. Using a computer-generated simple random sampling method, a total of 626 medical records were then selected from the combined pool of patients enrolled in these hospitals. Finally, we obtain 572 medical records that were included in the final analysis after excluding 18 fatal medical records and 36 incomplete medical records. Figure 1.

To ensure data quality, several measures were taken. First, the data extraction tool was checked for completeness and the existence of relevant variables through a preliminary chart review of 10% of the sample size at Kemisse General Hospital. The data collectors underwent training on the data collection process before conducting the actual data collection.

The supervisor continuously monitored the completeness, accuracy, and consistency of data collection throughout the data collection period. Finally, all completed data were examined for completeness and consistency before analysis.

### Statistical analysis

The data was entered using Epi-data Manager version 4.6. Analysis was conducted using Stata version 17, incorporating Kaplan-Meier curves and the log-rank test to evaluate survival status and associations. The bivariable analysis identified variables for inclusion in the multivariable analysis (p ≤ 0.25). Cox proportional hazard analysis was employed to estimate effect sizes, with statistical significance set at p ≤ 0.05. The fitness of the Cox proportional hazard model was assessed using Schoenfeld residuals and global tests, with insignificant p-values indicating a good fit.

### Ethical approval

Approval for ethical considerations was granted by the Ethical Review Committee of Wollo University, assigned with IRB number CMHS/03/14. Once approval was received, official letters were sent to the hospital’s quality unit control and approval for the study was secured. Confidentiality was maintained, with data restricted solely to the principal investigator. The study adhered strictly to the ethical principles outlined in the Declaration of Helsinki for medical research.

## Results

### Socio-demographic characteristics of the study participants

Among 572 study participants, 302(52.8%) were male, and 370(64.7%) were rural in residency. Regarding age distribution, the median age of the study participants was 45 years, and the mean age was 45.8 ± 14.1 SD years (Table 1).

**Table 1:** Socio-demographic characteristics of adult heart failure patients at selected South Wollo General Hospitals in 2022

Variables	Category	Status		Total (%)
		Readmitted (%) (n = 151)	No- **Readmitted** (%) (n = 421)	
Age	< 65	103(68.21)	388(92.16)	491(85.84)
	≥ 65	48(31.79)	33(7.84)	81(14.16)
Sex	Male	81(53.64)	221(52.49)	302(52.80)
	Female	70(46.36)	200(47.51)	270(47.20)
Residency	Urban	37(24.50)	165(39.19)	202(35.31)
	Rural	114(75.50)	256(60.81)	370(64.69)

### Clinical features of the study participants at index admission

When we see the clinical future of the study participants, almost one-third of 367 (64.16%) of the study participant are classified under NYHA class III, and the majority of 486 (84.97%) heart failure patients were discharged from the hospital within 7 days (Table 2).

**Table 2:** Clinical features of the study participants at index admission in heart failure patients with at selected South Wollo general Hospitals; Ethiopia, 2022

Variables	Category	Status		Total (%) (n = 572)
		Readmitted (%) (n = 151)	No-**Readmitted** (%) (n = 421)	
NYHA	II	5(3.31)	33(7.84)	38 (6.64)
	III	67(44.37)	300(71.26)	367 (64.16)
	IV	79(52.32)	88(20.90)	167 (29.20)
Length of hospital stay	<=7	73(48.34)	413(98.10)	486 (84.97)
	> 7	78(51.66)	8(1.90)	86(15.03)

### Treatment-related characteristics of study participants

During the index admission, almost two-thirds of 67.83% of participants had received diuretics, whereas 14.69% had received digoxin at discharge. During discharged at index admission diuretics, angiotensin-converting enzyme inhibitors (ACEIs) and beta-blockers were the most frequently prescribed medication (67.83%, 63.81% and 61.71%), respectively (Table 3).

**Table 3:** Treatment-related characteristics of study participants in heart failure patients with at selected South Wollo general Hospitals, Ethiopia, 2022

Variables	Category	Status		Total (%) (n = 572)
		Readmitted (%) (n= 151)	No- Readmitted (%) (n = 421)	
Digoxin	No	89(58.94)	399(94.77)	488(85.31)
	Yes	62(41.06)	22(5.23)	84(14.69)
Diuretics	No	19(12.58)	165(39.19)	184(32.17)
	Yes	132(87.42)	256(60.81)	388(67.83)
ACEI	No	73 (48.34)	134(31.83)	207(36.19)
	Yes	78(51.66)	287(68.17)	365(63.81)
ARB	No	100(66.23)	285(67.70)	385(67.31)
	Yes	51(33.78)	136(32.30)	187(32.69)
BB	No	98(64.90)	121(28.74)	219(38.29)
	Yes	53(35.10)	300(71.26)	353(61.71)
Vasodilators	No	134(88.74)	383(90.98)	517(90.38)
	Yes	17(11.26)	38(9.03)	55(9.62)
Anti-platelet	No	74(49.01)	241(57.25)	315(55.07)
	Yes	77(50.99)	180(42.76)	257(44.93)
Statin	No	141(93.38)	379(90.02)	520(90.91)
	Yes	10(6.62)	42(9.98)	52(9.09)

### The incidence of unplanned hospital readmission in heart failure patients

**Fig. 2:**
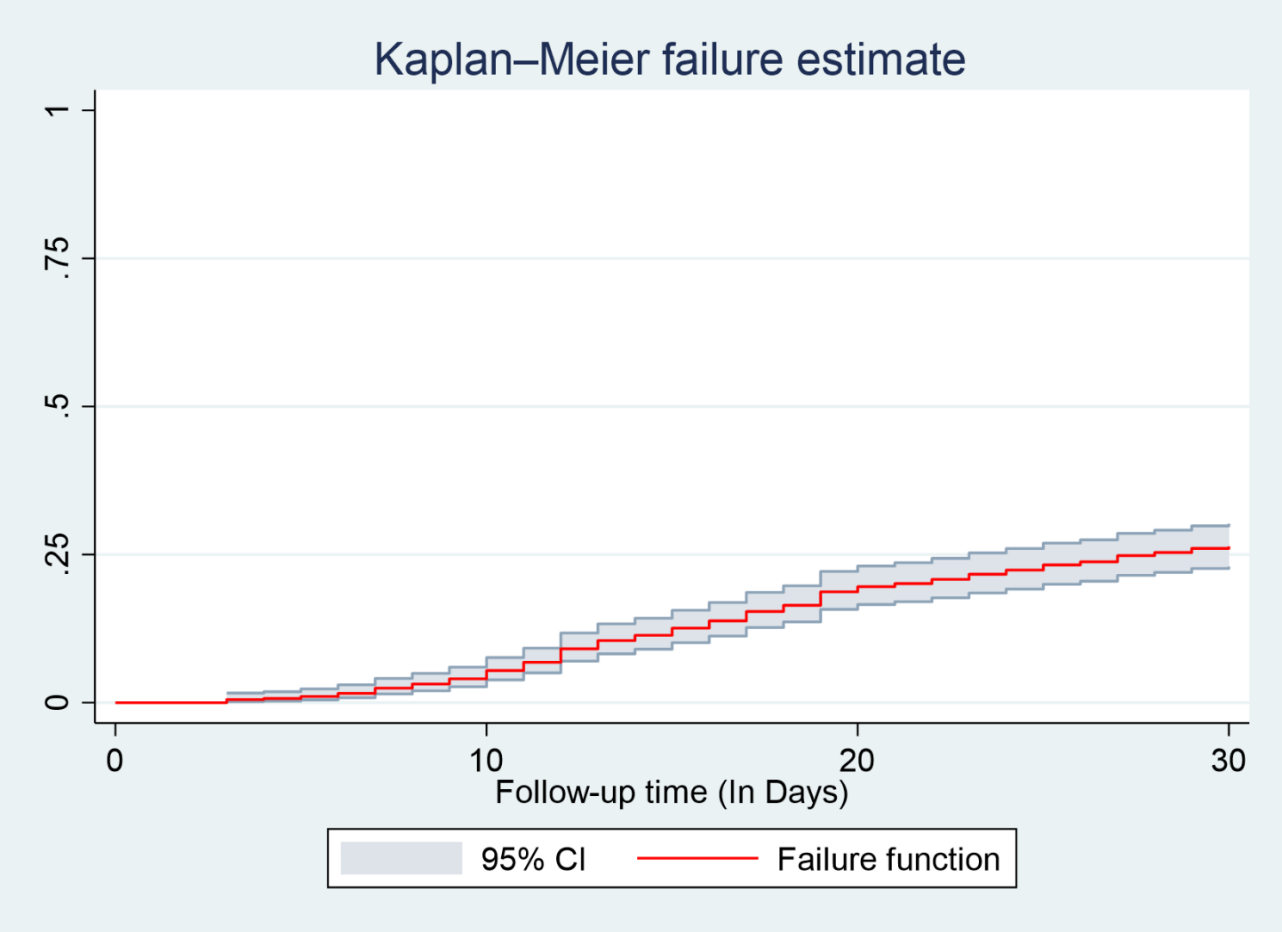
The overall Kaplan-Meier analysis of the hazard of unplanned readmission in heart failure patients at selected South Wollo general Hospitals; Ethiopia, 2022

In this study, 572 adult heart failure patients were followed retrospectively. The median time of readmission was 16 days (95% CI: 14, 17) with a minimum and maximum follow-up time of 3 and 30 days. In this study, 421 patients were censored, and 151 of them were readmitted within 30 days of discharge, resulting in a cumulative incidence of readmission of 26.40% (95% CI: (23.0, 30.2) during the follow-up period. The total follow-up time was 15,003 person-day, with an incidence rate of 10.06 readmission per 1000 person-day observations (95% CI: 8.59, 11.81) (Table 4). Figure 2.

**Table 4:** Person-time features of study participants in heart failure patients with at selected South Wollo general Hospitals, Ethiopia, 2022

Cohort in days	Person-time	Failures	Rate	[95% CI]
(0 - 5]	2838	6	0.00211416	(0.0009498–0.0047059)
(5 - 10]	2775	25	0.00900901	(0.0060875–0.0133327)
(10 - 15]	2598	41	0.01578137	(0.0116201–0.0214329)
(15 - 20]	2405	40	0.01663202	(0.0122–0.0226742)
(20 - 25]	2247	21	0.00934579	(0.0060935–0.0143339)
(30 - 25]	2140	18	0.00841121	(0.0052994–0.0133502)
Total	15,003	151	0.01006465	(0.0085808–0.0118051)

### Kaplan- Meier hazard estimation of unplanned readmission in heart failure patients

The overall Kaplan- Meier estimate showed that the hazard of unplanned hospital readmission of heart failure patients is low during the first three days following discharge after index admission. However, which relatively increases the hazard of unplanned readmission as follow-up time increases.

**Fig. 3:**
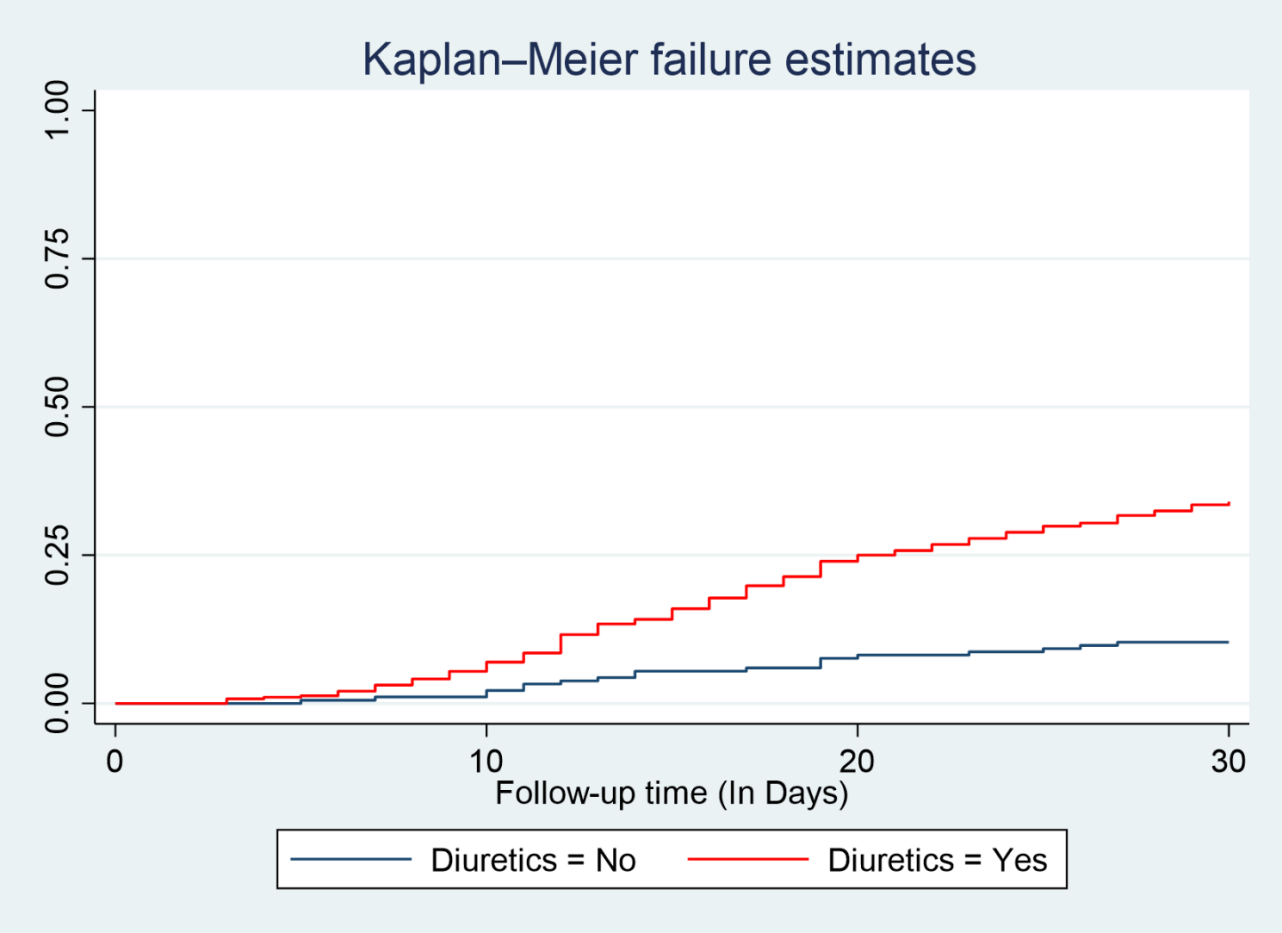
Kaplan- Meier hazard estimation of unplanned readmission in heart failure patients with categories of Diuretics use at selected South Wollo general Hospitals; Ethiopia, 2022

**Fig. 4:**
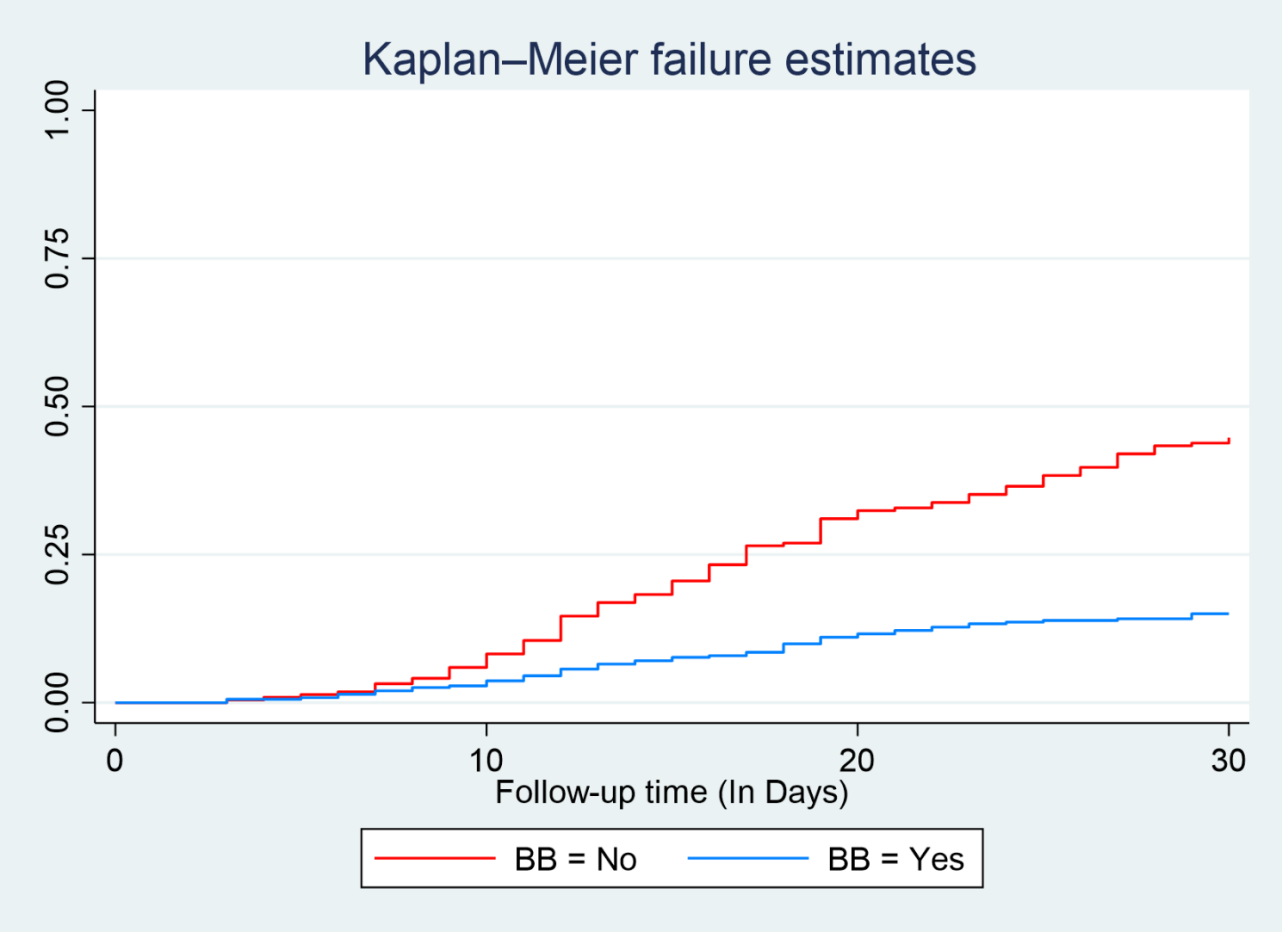
Kaplan- Meier hazard estimation of unplanned readmission in heart failure patients with categories of Beta Blockers use at selected South Wollo general Hospitals; Ethiopia, 2022

During follow-up time following discharge from the hospital during index admission to 30 days, the hazard curve tends to rise rapidly, implying unplanned reemitted in heart failure patients within this period. The hazard of readmission in Heart failure patient’s estimates varied depending on age, residency, length of hospital stay during index admission, a drug taken such as diuretics, and beta blockers at discharge were obtained. Figure 3, and Fig. 4.

### Predictors of unplanned hospital readmission in heart failure patients

Cox proportional hazard regression model was computed to identify the relationship between hazard of readmission and independent variables. In the Bi-variable Cox-Proportional Hazards regression model; sex, age, length of hospital stay (LOS), NYHF heart failure functional classification, ACEI, digoxin, diuretics, and beta Blockers were found to be p-value of less than 0.25 with unplanned hospital readmission. Those variables having a p-value of < 0.25 in the Bi-variable analysis were fitted in multivariable analysis.

In the multivariable cox proportional hazards model; age, LOS, diuretics, and Beta Blockers were Significant predictors of unplanned hospital readmission with a P-value of < 0.05.

The multivariable Cox-Proportional Hazards analysis revealed that in patients with heart failure, who were age ≥ 65 years had more than twofold hazard of unplanned readmission (AHR: 2.34, 95%CI: 1.63, 3.37) than those heart failure patients in the age group between 18 and 64 years. The hazard of unplanned readmission among heart failure patients was 1.85 times (AHR: 1.85, 95%CI: 1.07, 3.20) higher for rural in residency than that of urban. Those patients stay more than 7 Days during index admission had 3.68 times higher (AHR: 3.68, 95%CI: 2.51, 5.39) than the hazard of unplanned readmission than that of patients stay 1–7 Days.

Those patients discharge with beta blocker (BB) during index admission were 52% times less likely the hazard of unplanned readmission (AHR 0.48, 95%CI: 0 0.34, 0.69) compared to patients discharge without beta blocker (Table 5).

**Table 5:** Cox proportional hazard regression model of the study participants at index admission in heart failure patients with at selected South Wollo general Hospitals; Ethiopia, 2022

Independent variables	Category	Status		cHR(95%CI	aHR(95%CI	*P*-value
		Readmitted	No-Readmitted			
Age	≤ 64	61	388	1	1	
	≥ 65	90	33	8.22(5.92, 11.42)	2.34(1.63, 3.37)	0.001
Residency	Urban	16	185	1	1	
	Rural	135	236	5.32(3.17, 8.93)	1.85(1.07, 3.20)	0.028
LOS	1–7 Days	73	413	1	1	
	> 7 Days	78	8	12.64(9.06,17.62)	3.68(2.51, 5.39)	0.001
Diuretics	No	19	165	1	1	
	Yes	132	256	3.76(2.33, 6.09)	2.37(1.45, 3.86)	0.002
BB	No	98	121	1	1	
	Yes	53	300	0.28 (0.20, 0.39)	0.48(0 0.34, 0.69)	0.001

## Discussion

In this study, those patients who stay more than seven days during index admission with heart failure increase the risk of readmission as compared to patients discharged before seven days. This finding is consistent with a study conducted in the United States [15], India [16], the United States [17], Canada [18], and Southern California [19]. A longer in-hospital LOS may be indicative of delays in the delivery of care resulting from low staff-to-patient ratios, incoherent care from a team of healthcare professionals, lack of access to medical supplies, and other organizational factors that prevent optimal care [20]. Alternatively, this association might reflect the severity of the disease or the time needed to manage multiple disease conditions.

This suggests that patients who required a longer LOS at index admission were more likely to be hospitalized again. Therefore, such as patients should be counseled on regular and frequent hospital visits, tailored treatments, and lifestyle changes. Although our results cannot infer a causal relationship between LOS and readmission, they have some important policy implications. However, our results don’t imply that the incidence of unplanned hospital readmission is reduced by shortening hospital stays unless this can be achieved through more aggressive and effective therapies that reduce the severity of HF in the hospital, thus allowing for earlier discharge.

According to this study, findings show that patients who received beta-blockers on discharge were less likely to be readmitted than non-users. This was consistent with a study conducted in North Sydney [21], Australia [22], and china [23]. The benefits of using beta-blockers in patients with heart failure are physiologically known, as they inhibit sympathetic nervous system activity, reduce the risk of disease progression, improve symptoms, and prolong survival [24]. This implies that the use of beta-blockers at the time of discharge in patients with heart failure to prevent readmission would be profitable in the absence of a clinical contraindication.

The present study found that patients who received diuretics on discharge were at increased risk of unplanned hospital readmission compared with non-users. These findings are consistent with the study done in the Netherlands [25], Dutch [26], Japan [27], and Korea [28]were found that patients who receive loop diuretics independently predictor hospital readmission. However, this is in contrast with the finding of previous studies in Washington [29], Illinois [30], and Saudi Arabia [31]that diuretics users are less risky for unplanned hospital readmission. This contrasting finding might be due to the Poor medication adherence of study participants in this study. This possible justification is supported by a study done in sub-Saharan Africa, compliance with diuretics is poor due to side effects [32], and, Poor medication adherence was associated with increased readmissions in Tanzania [33]. In Ethiopia, studies [34] show that most patients are being discharged without discharge education.

In this study, we found a significant positive association between the NYHA class and diuretic utilization, indicating that diuretic utilization increased by 1.46 times for each increment in the NYHA class. This result is consistent with previous studies that demonstrated that NYHA class reflects the degree of HF and the requirement for diuretic therapy [35, 36]. This implies that diuretic utilization is a marker of HF severity and a predictor of poor prognosis. Therefore, optimizing diuretic therapy is essential for improving the quality of life and survival of HF patients.

### Limitations

This study has following limitations: Due to the retrospective nature of the study and the lack of linkage between hospitals, patients discharged from one hospital during the index admission may be readmitted to other hospitals within 30 days, but these readmissions might not be recorded. As a result, the readmission rate could be underestimated in such circumstances. In addition, important variables like personal behaviors such as smoking, alcohol consumption, and chat chewing were not adequately recorded medical reports of the patients and thus not included in the analysis. Therefore, further research is necessary to address these limitations.

## Conclusion

This study revealed that a rural residence, elderly age, longer hospital stays during the index admission, discharge with diuretics, and absence of beta-blockers prescription are independent predictors of unplanned hospital readmission in patients with heart failure. Hence, addressing these factors can lower unplanned hospital readmissions, enhance patient outcomes, and optimize heart failure management.

## Data Availability

All data generated or analyzed during this study are included in this published article.

## Abbreviations

ACEI: Angiotensin-Converting-Enzyme Inhibitors
BB: Beta-Blocker
HF: Heart Failure
LOS: Length of Hospital Stay
SSA: Sub-Saharan Africa
